# Prevalence and determinants of early markers of kidney damage in homozygotic sickle cell patients in the DR Congo: Update

**DOI:** 10.1007/s00467-026-07304-8

**Published:** 2026-05-05

**Authors:** Dieumerci Betukumesu Kabasele, Michel Aloni, Arriel Makembi Bunkete, François Kajingulu, Paul Lumbala Kabuyi, Orly Kazadi, Mireille Solange Nganga Nkanga, Joseph Bodi Mabiala, Tite Mikobi, Benjamin Longo Mbenza, Pipo Michel Tshiasuma, Jean Lambert Gini Ehungu

**Affiliations:** 1 Department of Pediatrics, University of Kinshasa, Kinshasa, Democratic Republic of the Congo; 2 Department of Clinical Biology, University of Kinshasa, Kinshasa, Democratic Republic of the Congo; 3 Department of Cardiology, University of Kinshasa, Kinshasa, Democratic Republic of the Congo; 4 Department of Nephrology and Dialysis, University of Kinshasa, Kinshasa, Democratic Republic of the Congo; 5 Department of Nephrology and Dialysis, University hospital of French Guiana, Saint-Laurent-du-Maroni Site, Saint-Laurent-Du-Maroni, French Guiana France; 6 Department of Genetics, University of Kinshasa, Kinshasa, Democratic Republic of the Congo; 7 Department of Pulmonology, University of Kinshasa, Kinshasa, Democratic Republic of the Congo

**Keywords:** Sickle cell disease, Albuminuria, Hyperfiltration, Congolese children

## Abstract

**Background:**

Sickle cell nephropathy is a common complication of sickle cell disease that begins in childhood and can progress silently to chronic kidney disease. In the Democratic Republic of Congo (DRC), data on early kidney damage in children with sickle cell disease remain limited. In 2017, studies conducted in the same context reported separately on the prevalence of microalbuminuria and glomerular hyperfiltration (GHF). This study aimed to update the prevalence of albuminuria and GHF and to determine their associated factors in children with sickle cell disease in the DRC.

**Methods:**

We conducted a cross-sectional study including 175 children with sickle cell disease, followed up in four hospitals in Kinshasa. High albuminuria and GHF, the main evaluation criteria, were defined respectively by an albuminuria/creatinine ratio (ACR) ≥ 30 mg/g and an estimated glomerular filtration rate (eGFR) > 130 ml/min/1.73 m^2^ in girls and > 140 ml/min/1.73 m^2^ in boys, according to the Schwartz formula.

**Results:**

Among the 175 children included, 28.5% had high albuminuria and 38.3% had GHF. Factors significantly associated with early renal involvement were frequent blood transfusions (≥ 9/year), recurrent vaso-occlusive crises (≥ 3/year), low fetal hemoglobin levels (< 15%), and markers of hemolysis (LDH > 400 IU/L and elevated indirect bilirubin). These results reflect a high persistence of early renal impairment nearly nine years after the first data were published.

**Conclusions:**

Early markers of kidney damage remain very common in children with homozygous sickle cell disease in the DRC. This persistence highlights the lack of effective kidney prevention strategies and the urgent need for systematic screening using simple and accessible tools in resource-limited settings.

**Graphical Abstract:**

**Figure:**
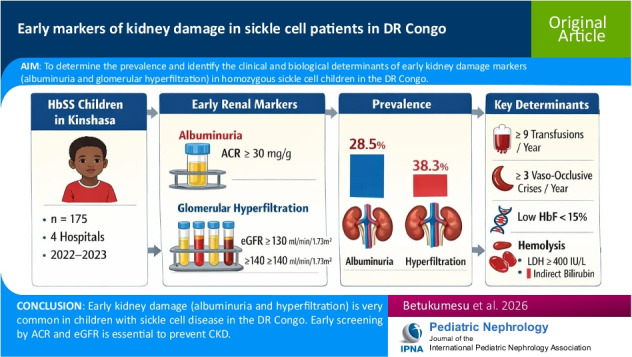
A higher resolution version of the Graphical abstract is available as Supplementary information

**Supplementary Information:**

The online version contains supplementary material available at 10.1007/s00467-026-07304-8.

## Introduction

Sickle cell disease is the most common genetic disorder in the world and remains highly prevalent in the Democratic Republic of Congo (DRC), one of the countries most affected [1–3]. Children with homozygous sickle cell disease are exposed early on to numerous complications, particularly renal complications, which can silently progress to chronic kidney disease and contribute to early mortality [1, 4–8]. Homozygous sickle cell disease (HbSS) is characterized by erythrocyte abnormalities that cause microvascular obstruction, chronic hemolysis, and multivisceral damage, unlike the generally more benign heterozygous forms [1, 9, 10].

The associated renal involvement, known as sickle cell nephropathy, results from complex mechanisms involving chronic hypoxia, medullary ischemia, oxidative stress, endothelial dysfunction, and genetic factors, particularly *APOL1*, *MYH9*, and *HMOX* [6, 11–19]. Sickle cell nephropathy is one of the most common and severe chronic complications of sickle cell disease. Kidney damage often begins in childhood and manifests early as glomerular hyperfiltration (GHF) and albuminuria, which can gradually progress to chronic kidney disease and then kidney failure in adulthood [16, 20, 21]. In resource-limited settings, where access to renal replacement therapies is extremely limited, early identification of kidney damage is essential in order to implement appropriate preventive interventions [22].

In 2017, Aloni et al. separately reported the prevalence of microalbuminuria and GHF in children with homozygous sickle cell disease in Kinshasa, DRC [21, 23, 24]. Nearly nine years later, there are no updated data available to assess the evolution of these early markers in this population. The aim of the present study was therefore to provide an update on the prevalence of albuminuria and GHF, as well as to identify the factors associated with them in children with HbSS in the DRC, in order to guide screening and management strategies adapted to resource-limited settings.

## Methods

### Type and setting of the study

This is a cross-sectional study conducted between August 2022 and August 2023 in four hospitals in the city of Kinshasa that specialize in the care of patients with sickle cell disease. These include the Diamond Medical Center, the Center for Mixed Medicine and Anemia (CMMAS), the Biamba Marie Mutombo Hospital, and the University Clinics of Kinshasa.

### Study population

Children aged 2 to 18 years with confirmed HbSS were recruited consecutively during follow-up consultations. We obtained the free and informed consent of parents or guardians and the assent of children. Patients with acute illness at the time of inclusion, chronic kidney disease unrelated to sickle cell disease, or incomplete clinical or biological data were excluded. Children receiving treatment with angiotensin-converting enzyme (ACE) inhibitors, angiotensin II receptor antagonists (ARA II), and/or hydroxyurea (HU) were also excluded, as these drugs may have an impact on proteinuria.

A total of 175 children were included in the final analysis.

### Data and sample collection

The following formula was used to estimate the minimum size of the study population:

$$n=Z^2\left(pq\right)/d^2$$

where *n* = sample size, Z = 95% confidence level, *p* = proportion of the target population with microalbuminuria (18%), q = proportion of the target population without this characteristic (0.82), and d = precision level (0.05). The prevalence of 18%, recently reported by Aloni et al. [21] in children with sickle cell disease in the DRC, was used as the reference value for this study. Therefore, q = (1–0.18) = 0.82. The minimum population size was therefore estimated at 227 participants. In the present study, 52 children were excluded: 37 after electrophoretic testing and 15 others due to incomplete clinical and biological data. Thus, only 175 children with confirmed sickle cell disease participated in the study.

Sociodemographic, clinical, and therapeutic data were collected using a standardized form. Clinical variables included age, sex, frequency of vaso-occlusive crises, transfusion history, and use of hydroxyurea. In the local context, repeated blood transfusions mainly reflect the management of severe anemia in children who do not have access to hydroxyurea and were considered an indirect marker of disease severity.

### Anthropometric measurements and blood pressure

Blood pressure was measured after at least five minutes of rest, using an age-appropriate cuff. Blood pressure categories were defined according to the recommendations of the American Academy of Pediatrics (2017) [25] (Table 1).

**Table 1:** Blood pressure categories and definition of hypertension

Children under 13 years old	Children over 13 years old
Normal BP: < 90th percentile	Normal BP: < 90th percentile
Elevated BP: ≥ 90th percentile to < 95th percentile or 120/80 mmHg to < 95th percentile	High BP: ≥ 90th percentile to < 95th percentile or 120/80 mmHg to < 95th percentile
Stage 1 hypertension: ≥ 95th percentile to < 95th percentile + 12 mmHg or 130/80 to 139/89 mmHg	Stage 1 hypertension: ≥ 95th percentile to < 95th percentile + 12 mmHg or 130/80 to 139/89 mmHg
Stage 2 hypertension: ≥ 95th percentile + 12 mmHg or ≥ 140/90 mmHg	Stage 2 hypertension: ≥ 95th percentile + 12 mmHg or ≥ 140/90 mmHg

Source: Flynn et al. 2017 [25]

### Biological tests

Venous blood samples were taken to measure hemoglobin (Hb), bilirubin, lactate dehydrogenase (LDH), and fetal hemoglobin (HbF). The analyses were performed at the laboratory of the Faculty of Medicine of the University of Kinshasa using a Minicap® automated analyzer (Sebia, Phoresis Rel 8.6.3.).

Renal assessment was based on the measurement of the urinary albumin/creatinine ratio (ACR) obtained from a spot urine sample and analyzed by immunoturbidimetry. Albuminuria was defined as a ratio ≥ 30 mg/g. In accordance with KDIGO recommendations, ACR was classified as follows: normal albuminuria grade A1 (ACR < 30 mg/g), albuminuria grade A2 (ACR 30–299 mg/g), and albuminuria grade A3 (ACR ≥ 300 mg/g) [26].

Creatinine was measured using an enzymatic method with a Cobas C111® automated analyzer (Roche Instrument Center, Rotkreuz, Switzerland).

Estimated glomerular filtration rate (eGFR) was calculated using the Schwartz formula, and GHF was defined as a rate > 130 mL/min/1.73 m^2^ in girls and > 140 mL/min/1.73 m^2^ in boys. Renal failure was defined as an GFR < 60 mL/min/1.73 m^2^.

### Statistical analysis

The data collected were entered into Excel 2024® and then analyzed using SPSS 25.0 software. Results are presented in the form of tables and graphs. For qualitative variables, we determined frequencies and percentages, while for quantitative variables we used measures of central tendency (mean and median) and dispersion (standard deviation; interquartile range). Proportions were compared using the Chi-square test or Fischer's exact test (as appropriate). Student’s t-test, Mann–Whitney U-test, Pearson's Chi-square test or Fisher's exact test were used to compare independent groups, where appropriate. The logistic regression test was used to search for factors associated with early markers of renal impairment, with calculation of the Odds Ratio and 95% confidence intervals. For all tests used, *p* < 0.05 was the threshold for statistical significance.

### Ethical considerations

The study protocol was approved by the National Health Ethics Committee of the Ministry of Public Health, Hygiene, and Prevention of the Democratic Republic of Congo to ensure compliance (Approval reference: No. 385/CNES/BN/PMMF/2022 dated September 19, 2022).

## Results

### Characteristics and description of the study population

A total of 175 children with HbSS were included in the final analysis. All were being monitored at one of four specialized pediatric centers in Kinshasa and were clinically stable at the time of assessment.

Table 2 presents the sociodemographic and clinical characteristics of the population by gender. The sample included 91 girls (52%) and 84 boys (48%), with an overall mean age of 9 ± 4 years. The mean age differed significantly between the two sexes: 9 ± 4 years for girls versus 10 ± 4 years for boys (*p* = 0.003). The annual median number of vaso-occlusive crises was 1.0 (IQR: 1.0–2.0) and that of transfusions was 4.0 (IQR: 3.0–5.0). With the exception of age, none of the other characteristics compared between the sexes showed a statistically significant difference (*p* > 0.05).

**Table 2:** Sociodemographic and clinical characteristics of population by sex

Variables	Population *N* = 175	Female *N* = 91	Male *N* = 84	P
Age, years (X ± SD)	9 ± 4	9 ± 4	10 ± 4	0.003
2–5 years, n (%)	37 (21)	24 (25.2)	13 (15.5)	
6–10 years, n (%)	70 (40)	36 (37.9)	34 (40.47)	
11–15 years, n (%)	57 (33)	23 (27.3)	34 (40.47)	
> 15 years, n (%)	11 (6)	8 (8.4)	3 (3.57)	
Weight, kg (X ± SD)	24 ± 11	24 ± 11	24 ± 10	0.829
Height, cm (X ± SD)	121 ± 22	122 ± 22	119 ± 23	0.298
BMI, kg/m^2^ (X ± SD)	17 ± 8	16 ± 6	18 ± 9	0.101
SaO2, %(X ± SD)	97 ± 1.6	96 ± 1.3	96 ± 1.9	0.769
VOC/year (%)	1.0(1.0–2.0)	1.0 (1.0–2.0)	1.0 (1.0–3.0)	0.812
None, n (%)	36(20.60)	14(15.4)	22(26.2)	0.203
(1–2), n (%)	69 (39)	39(42.9)	30 (35.7)	
≥ 3, n (%)	70(40)	38(41.8)	32(38)	
Transfusion/year (IQR)	4.0 (3.0–53.0)	4.0(3.0–5.0)	4.0 (3.30–4.0)	0.278
None, n (%)	8(4.6)	1(1.1)	7(8.3)	0.067
1–8, n (%)	127(72.6)	66(72.5)	61(72.6)	
9–16, n (%)	31(17.7)	20(22.0)	11 (13.1)	
≥ 16, n (%)	9(5.1)	4(4.4)	5(6.0)	
SBP (X ± SD) mmHg	102.9 ± 17.3	104.5 ± 13.6	100.8 ± 21.6	0.582
DBP (X ± SD) mmHg	67.8 ± 9.4	68.6 ± 8.9	66.6 ± 10.3	0.127

Data are expressed as mean ± standard deviation, median (interquartile range), absolute frequency (n) and relative frequency (percent). *VOC* vaso-occlusive crisis, *SDB* systolic blood pressure, *DBP* diastolic blood pressure, *BMI* body mass index

### Biological characteristics

Table 3 shows the biological characteristics of the study population by gender.

**Table 3:** Biological characteristics of homozygous sickle cell patients according to sex

Variables	Population *N* = 175	Female *N* = 91	Male *N* = 84	P
**Renal parameters**				
ACR mg/g (X ± SD)	128 ± 59.1	116.3 ± 44.4	145.3 ± 71.7	0.000
Albuminuria grade A1, n (%)	125(71.4)	64(70.32)	61(72.61)	0.201
Albuminuria gra1de A2, n (%)	41(23.4)	22(24.17)	19(22.61)	0.391
Albuminuria grade A3, n (%)	9(5.1)	5(5.49)	4(4.76)	0.549
eGFR (ml/min/1.73 m^2^) (X ± SD)	130 ± 58	129 ± 64	131 ± 60	0.811
Hyperfiltration, n (%)	67(38.3)	35(38.46)	32(38.09)	0.322
Failure, n (%)	7(4)	4(4.3)	3(3.57)	0.202
**Hematological parameters**				
Hb g/dl (X ± SD)	7 ± 1	7 ± 1	7 ± 1	0.192
HbF % (EIQ)	9.7 (8.6–10.7)	8.6(7.3–9.9)	10.9 (9.5–13.6)	0.019
LDH IU/L (X ± SD)	1646 ± 686	1637 ± 636	1656 ± 741	0.851
BI > 1 mg/dl (IQR)	0.78(0.63–0.87)	0.68(0.51–0.76)	0.68(0.53–0.87)	0.567
BT, mg/dl (IQR)	1.5(1.4–1.6)	2.4 (2.0–2.8)	1.7 (1.5–2.0)	0.014
BT > 2 mg/dl, n (%)	54(31)	35(37.4)	19 (23.75)	0.030
ALAT, IU/L (X ± SD)	22 ± 15	21 ± 14	24 ± 15	0.322
ASAT, IU/L (X ± SD)	50 ± 25	49 ± 22	51 ± 27	0.595

Data are presented as mean ± SD, median (IQR), or n (%). Albuminuria was defined according to KDIGO as A1 (ACR < 30 mg/g), A2 (30–299 mg/g), and A3 (≥ 300 mg/g). Hyperfiltration was defined as eGFR > 130 mL/min/1.73 m^2^ in girls and > 140 mL/min/1.73 m^2^ in boys, and failure as eGFR < 60 mL/min/1.73 m^2^

*ACR* albumin-to-creatinine ratio, *eGFR* estimated glomerular filtration rate, *Hb* hemoglobin, *HbF* fetal hemoglobin, *LDH* lactate dehydrogenase, *BI* indirect bilirubin, *BT* total bilirubin *ALAT* alanine aminotransferase, *ASAT* aspartate aminotransferase

**Fig. 1:**
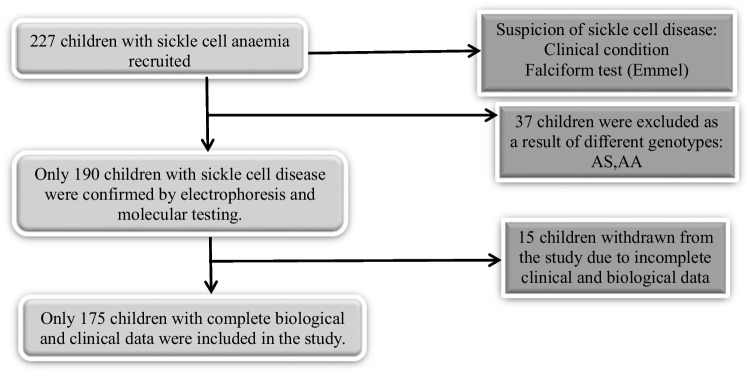
Organisational chart of the population studied

The mean values for ACR and eGFR for the entire group were 128 ± 59.1 mg/g and 130 ± 58 ml/min/1.73 m^2^ respectively. Albuminuria was observed in 50 patients (28.5%), with grade A2 albuminuria and grade A3 albuminuria in 41 patients (23.4%) and 9 patients (5.1%), respectively. Hyperfiltration was present in 67 patients (38.3%). Mean LDH and median total bilirubin, markers of hemolysis, were 1646 ± 686 IU/l and 1.5 (1.4–1.6) mg/dl, respectively. Median HbF was 9.7 (8.6–10.7) %, with 10.9 (9.5–13.6) % in boys and 8.6 (7.3–9.9) % in girls (*p* = 0.019). In contrast, the median total bilirubin [2.4 (2.0–2.8) vs. 1.7 (1.5–2.0) mg/dl; *p* = 0.014] and the proportion of children with total bilirubin > 2 mg/dl [37.4% vs. 23.75%; *p* = 0.030] were significantly higher in girls than in boys. The differences observed for the other variables of interest between the two sexes were not statistically significant (Table 3) (Fig. 1).

### Early markers of renal impairment in the study population

**Fig. 2:**
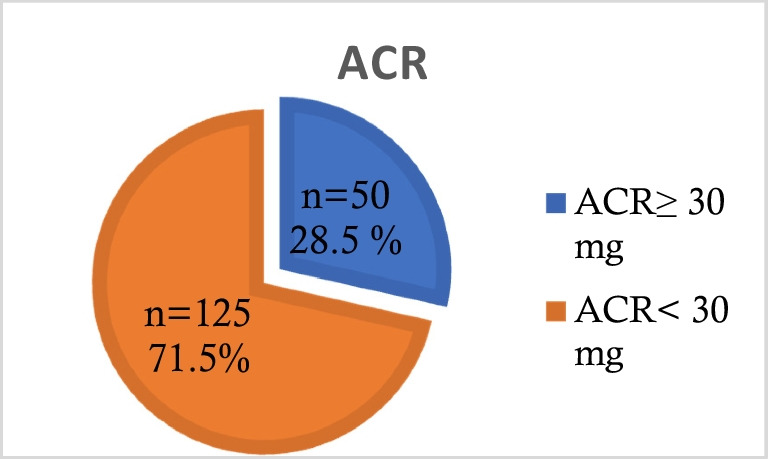
Frequency of albuminuria in homozygous sickle cell patients

**Fig. 3:**
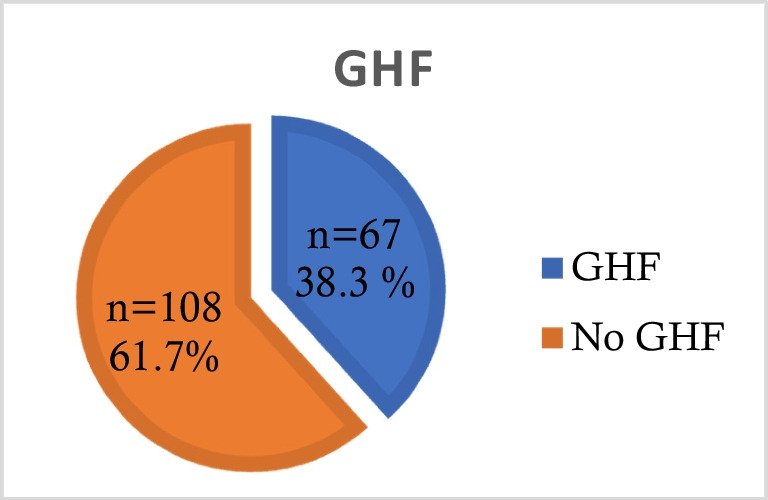
Frequency of glomerular hyperfiltration in homozygous sickle cell patients

In our series, out of a total of 175 homozygous sickle cell patients, only 50 patients (28.5%) had albuminuria (Fig. 2), and GHF was present in 67 patients (38.3%) (Fig. 3).

### Sociodemographic, clinical and biological characteristics of population according to albuminuria

Fifty patients (28.5%) had albuminuria. Their mean age, SBP and DBP were 9 ± 4 years, 105.9 ± 16.1 mmHg and 68.7 ± 10.5 mmHg respectively. The proportion of children with more than 3 vaso-occlusive crises (VOCs) /year and more than 9 transfusions/year and albuminuria was 26 (52%) and 15 (30%), respectively. Patients with increased albuminuria had a higher proportion of VOCs and blood transfusions, with a statistically significant difference observed (*p* = 0.034; *p* = 0.022). Hyperfiltration and decreased eGFR were observed in 29 (58%) and 4 (8%) patients with albuminuria, but the difference observed was not statistically significant (*p* = 0.501; *p* = 0.315). Compared with patients with grade A1 albuminuria (normal albuminuria), those with increased albuminuria (grades A2, A3) had a lower proportion of fetal hemoglobin (< 15%), elevated levels of LDH and indirect bilirubin, markers of hemolysis (Table 4).

**Table 4:** Clinical and biological characteristics of population by early markers

Variables	ACR < 30 mg/g (*n* = 125)	ACR ≥ 30 mg/g (*n* = 50)	p	No HFG (*n* = 108)	HFG (*n* = 67)	p
Age (yr)	9 ± 4	9 ± 4	0.728	10 ± 4	9 ± 4	0.639
Sex (M/F)	43/82	40/10	0.019	45/63	37/30	0.230
Wt (kg)	24 ± 11	24 ± 11	0.863	24 ± 10	24 ± 11	0.635
Ht (cm)	120 ± 22	123 ± 23	0.274	120 ± 22	123 ± 23	0.274
BMI (kg/m^2^)	17 ± 7	17 ± 8	0.426	17 ± 7	16 ± 8	0.707
SBP/DBP (mmHg)	103/62	106/69	0.260	104/65	104/65	0.551
VOC ≥ 3/yr, n (%)	47 (37.6)	26 (52)	0.034	19 (18.8)	16 (23.9)	0.778
Transf ≥ 9/yr, n (%)	17 (13.6)	15 (30)	0.022	75 (74.3)	46 (68.7)	0.151
SaO₂ (%)	96 ± 2	96 ± 2	0.907	97 ± 1	96 ± 2	0.499
ACR, mg/g	—	—	—	14.8 (7.0–62.9)	54.6 (39.7–148.5)	< 0.001
eGFR, n (%)	65 (55–76)	101 (91–161)	0.705	46 (41–49)	125 (118–130)	< 0.001
HFG, n (%)	38 (30.4)	29 (58)	0.501	—	—	—
Failure (%)	3 (2.4)	4 (8)	0.315	—	—	—
HbF < 15%, n (%)	94 (75.2)	41 (82)	0.013	76 (70.4)	58 (86.6)	0.020
Hb (g/dL)	7.1 ± 1	6.9 ± 1.2	0.508	7.0 (6.6–7.8)	7.0 (6.6–7.5)	0.127
LDH, IU/L (X ± SD)	1566 ± 699	1796 ± 630	0.029	1656 ± 738	1576 ± 589	0.088
LDH > 400 IU/L, n (%)	69 (55.2)	39 (78)	0.020	58 (53.7)	38 (56.7)	0.071
BI > 1 mg/dL, n (%)	84 (67.2)	40 (80)	0.048	69 (63.8)	52 (77.6)	0.018
BD > 1 mg/dL, n (%)	20 (16)	9 (18)	0.463	20 (18.5)	7 (10.4)	0.167
BT > 2 mg/dL, n (%)	37 (29.6)	17 (34)	0.272	33 (30.5)	21 (31.3)	0.394
ASAT (IU/L)	51 ± 26	47 ± 21	0.645	42 (36–63)	39 (36–58)	0.622
ALAT (IU/L)	20 ± 12	17 ± 12	0.053	17 (12–28)	17 (12–25)	0.191

Data are expressed as mean ± SD, median (IQR), or n (%)

*ACR* albumin-to-creatinine ratio, *HFG* hyperfiltration defined as eGFR > 130 mL/min/1.73 m^2^ in girls, > 140 in boys. *Failure* eGFR < 60 mL/min/1.73 m^2^. Wt, weight; Ht, height, *BMI* body mass index, *SBP* systolic, *BP* *DBP* diastolic, *BP* *VOC* vaso-occlusive crisis, *Transf* transfusions, *SaO₂* oxygen saturation, *eGFR* estimated glomerular filtration rate (mL/min/1.73 m^2^), *Hb* hemoglobin, *HbF* fetal hemoglobin, *LDH* lactate dehydrogenase, *BI* indirect bilirubin, *BD* direct bilirubin, *BT* total bilirubin, *ALAT* alanine aminotransferase, *ASAT* aspartate aminotransferase

### Sociodemographic, clinical and biological profile of population based on glomerular hyperfiltration

As shown in Table 4, 67 patients (38.3%) had hyperfiltration in the present study. Their mean age was 9 ± 4 years. The proportion of children with multiple blood transfusions (≥ 9), HbF < 15%, indirect bilirubin (BI) > 1 mg/dl and LDH was 68.7%; 86.6%; 77.6%, respectively. Median eGFR was 124.8 (117.9–129.9) ml/min/1.73 m^2^. The differences observed for all these variables were statistically significant. The median urinary ACR was significantly higher in the group with GHF than in the group without hyperfiltration, suggesting a partial overlap between albuminuria and GHF (Table 4).

### Determinants of albuminuria

Table 5 presents an analysis of the determinants of albuminuria. Factors associated with increased albuminuria (ACR ≥ 30) in sickle cell patients, in univariate analysis, were: male gender, number of VOCs ≥ 3/year, number of transfusions ≥ 9/year and HbF < 15%. After adjustment, number of transfusions ≥ 9/year, BI > 1 mg/dl, LDH > 400 IU/L and HbF < 15% were found to be independent determinants of an ACR ≥ 30, with risk multiplied by 3 for number of transfusions ≥ 9/year, 5 for BI > 1 mg/dl, 2 for LDH > 400 IU/L and HbF < 15%.

**Table 5:** Logistic regression determinants of pathological albuminuria in homozygous sickle cell children

Variables	Univariate analysis		Multivariate analysis	
	P	OR (95% CI)	P	ORa (95% CI)
Sex				
Female		1		1
Male	0.042	2.11 (1.59–2.62)	0.406	1.49 (0.58–3.86)
VOC/year				
None		1		1
1–2	0.630	0.80 (0.32–1.96)	0.479	1.72 (0.38–3.44)
≥ 3	0.034	1.55 (1.14–3.53)	0.049	1.91 (1.24–6.36)
Transfusion/year				
None		1		1
1–8	0.919	1.21 (0.35–1.96)	0.925	1.06 (0.21–1.68)
≥ 9	0.022	4.12 (1.47–6.25)	0.019	3.49 (1.46–7.44)
HbF				
≥ 15		1		1
< 15	0.013	2.60 (1.56–4.53)	0.041	2.64 (1.50–5.37)
BI > 1 mg/dl, n (%)	0.048	9.89 (1.98–18.0)	0.041	5.80 (1.62–11.4)
LDH > 400 IU/L	0.029	1.73 (0.76–4.33)	0.036	2.710 (1.04–6.72)

Data are expressed as mean ± standard deviation, median (interquartile range), absolute (n) and relative (percent) frequency. *VOC* vaso -occlusive crisis, *LDH* lactate dehydrogenase, *BI* indirect bilirubin

### Determinants of glomerular hyperfiltration

In univariate logistic regression analysis, male gender, number of transfusions ≥ 9/year, BI > 1 mg/dl and HbF < 15% were the factors associated with GHF. In multivariate analysis, the emerging factors independently associated with GHF were: number of transfusions ≥ 9/year with a fourfold increased risk; for BI > 1 mg/dl and HbF < 15%, the risk was twofold (Table 6). Data are expressed as mean ± standard deviation, median (interquartile range), absolute frequency (n) and relative frequency (as a percentage).

**Table 6:** Logistic regression determinants of glomerular hyperfiltration in children with homozygous sickle cell disease

Variables	Univariate analysis			Multivariate analysis	
	P	OR (95% CI)		P	ORa (95% CI)
Sex					
Female		1			1
Male	0.031	1.78 (1.05–3.04)		0.246	1.56 (0.73–3.30)
Transfusion					
None		1			1
1–8	0.707	1.50 (0.18–2.46)		0.132	0.24 (0.04–1.53)
** ≥ 9**	0.016	6.80 (1.44–10.33)		0.037	4.11 (1.15–8.77)
HbF					
** ≥ 15**		1			
** < 15**	0.031	2.57 (1.09–6.06)		0.015	2.27 (1.84–6.09)
BI > 1 mg/dl	0.018	0.98(0.96–0.99)		0.045	1.54(0.99–2.08)

Data are expressed as mean ± standard deviation, median (interquartile range), absolute (n) and relative (percent) frequency. *HbF* Fetal hemoglobin, *BI* Indirect bilirubin

## Discussion

In children with sickle cell disease, hyperfiltration and albuminuria are more common during early childhood and adolescence [27]. To our knowledge, few studies have reported renal dysfunction, particularly alterations in early markers of renal damage, in children with homozygous sickle cell disease in the Democratic Republic of Congo. This study examines the prevalence and determinants of early renal markers (albuminuria and GHF) in these children. In the present study, grade A2 albuminuria (23.4%) and grade A3 albuminuria (5.1%) were observed, representing a 28.5% increase in albuminuria. The prevalence of increased albuminuria varies between studies.

Several studies conducted in children and adults with sickle cell disease have shown that the prevalence of albuminuria increases with age. This prevalence ranges from 4.5% to 26% in patients under the age of 21 [28] and from 26 to 68% in those aged 21 and over [28]. Our prevalence of high albuminuria is higher than the 11.8% and 18% reported by Itokua et al. [31] and Aloni et al. [21], respectively, in children with sickle cell disease in Kinshasa. A prevalence of 28%, similar to ours, was reported by Mawanda et al. in Uganda [29]. This prevalence is also comparable to the 30.4% observed by Mona Hassan Eltagui in Egypt [30]. However, this prevalence is lower than the respective rates of 86.7%, 60.9%, 50%, and 48.8% reported in adults and children with sickle cell disease by Kambale et al. in northeastern DRC [32], Geard et al. in Cameroon [19], Bolarinwa et al. in Nigeria [33], and Engole et al. [34] in Kinshasa. It is also lower than that observed in patients with sickle cell living outside Africa. In fact, grade A2 albuminuria was found in 51% of adult sickle cell patients by Kormann in France [16] and in 40% by Haymann in the United States [27]. The frequency of grade A3 albuminuria in the present study was 5.1%, higher than the 2.5% observed by Geard et al. [19] in adults and children with sickle cell disease. Outside Africa, the prevalence of grade A3 albuminuria in adult patients with sickle cell disease is 19%, 16.5%, and 22%, respectively, according to Haymann in the United States [27], Asnani in Jamaica [35], and Kormann in France [16].

The differences in prevalence observed between studies may be explained by methodological differences. Although these differences may reflect different sickle cell phenotypes, they could also result from variations in study populations, methodology, sample sizes, and criteria for defining albuminuria. For example, Kormann et al. [16] used thresholds of 3 mg/mmol and > 30 mg/mmol to define microalbuminuria and macroalbuminuria (grade A2 and grade A3, respectively), whereas Bolarinwa et al. [33] used thresholds of 30 μg/mg and > 300 μg/mg.

Approximately four out of ten children in this study had GHF (38.3%). This prevalence is higher than the 30% reported by Aloni et al. [24] in a similar context, and higher than the 19.9% and 22.4% reported by Kambale [32] and Engole et al. [34], respectively. However, it is lower than the 45.9% and 90% reported by Geard et al. [19] and Garba [36]. Differences in methodology, study populations, sample sizes, and thresholds used to define hyperfiltration likely explain the observed disparities. Except for Aloni’s study, which focused exclusively on children, most studies included both children and adults with sickle cell disease. In our study, only children were considered.

Although the cross-sectional nature of this study does not allow for the establishment of a formal temporal relationship, the higher prevalence of GHF compared to elevated ACR suggests that hyperfiltration may represent an earlier renal abnormality in sickle cell nephropathy. These results are consistent with those reported by Lebensburger et al., who showed that GHF precedes the onset of albuminuria in children with sickle cell disease [37]. In the same study, the authors also observed that patients with early hyperfiltration were more likely to develop early albuminuria [37]. This phenomenon could be explained by an abnormal increase in glomerular filtration rate, most often secondary to an increase in intraglomerular pressure, leading to an alteration of the glomerular filtration barrier and urinary albumin leakage. Thus, measuring eGFR from early childhood could enable early identification of patients with hyperfiltration and enhance screening for albuminuria at an earlier stage of the disease [37]. While albuminuria remains a simple and widely accessible screening marker, GHF could reflect an even earlier stage of kidney damage. These data support the complementary use of these two markers in strategies for the early detection of sickle cell nephropathy.

Regarding the determinants of early markers of sickle cell nephropathy, multiple transfusions, the number of VOCs, low HbF levels, and markers of hemolysis (BI and LDH) were the main factors associated with high albuminuria and GHF. The association with multiple transfusions and low HbF levels may be explained by underlying hemolysis, which contributes to both albuminuria and hyperfiltration. HbF is recognized as a modulating factor in sickle cell disease, preventing vaso-occlusive and hemolytic crises. Hydroxyurea is recommended as a first-line treatment for sickle cell disease due to its beneficial effects on HbF levels [38–40].

Chronic hemolysis may be a pathological feature explaining the high risk of GHF, proposed as the first stage of sickle cell nephropathy [41]. Prolonged hyperfiltration likely contributes to albuminuria and always precedes it [37]. Supporting this, Aloni et al. reported that 22% of their pediatric patients with moderate albuminuria had hyperfiltration [24].

Our study observed strong associations between hemolytic markers (BI and LDH) and early renal impairment. These findings are consistent with reports by Itokua et al. [31] in children and by Maier-Redelsperger [41] in adults. Day et al. also reported associations between elevated hemolysis markers and albuminuria and hyperfiltration in adults with HbSS, suggesting their potential as clinical indicators of nephropathy risk [42].

Sickle cell patients receiving multiple transfusions (> 9 per year) were four times more likely to develop high albuminuria. In our context, the frequency of blood transfusions appears to be a significant determinant of early kidney damage. In the DRC, repeated transfusions mainly reflect the management of severe anemia in patients who do not have access to hydroxyurea. Children who received ≥ 9 transfusions per year generally had more severe anemia and persistent hemolysis, a major pathophysiological mechanism involved in the development of sickle cell nephropathy. Thus, transfusion frequency could be an indirect marker of disease severity and a relevant clinical warning indicator in the nephrological follow-up of these patients. These data support the hypothesis that hemolysis plays a key role in the development of sickle cell nephropathy [6, 29]. In contrast, Alvarez et al. [43] reported a protective effect of transfusions against moderate albuminuria at chronic renal failure onset, likely because those patients received hydroxyurea, which reduces hemolysis.

VOCs and low HbF levels were also associated with albuminuria. Children experiencing > 3 VOCs per year had double the risk of albuminuria. HbF protects against hemoglobin polymerization and sickling, reducing vaso-occlusive events [44]. Vascular occlusion leads to cortical hyperperfusion and medullary hypoperfusion, contributing to renal lesions [6]. Vaso-occlusion may also trigger chronic coagulation and inflammation, with platelets secreting inflammatory cytokines such as IL-6, which play a role in albuminuria development [44].

In summary, the prevalence of early kidney damage markers (albuminuria and hyperfiltration) was 28.5% and 38.3%, respectively. The number of vaso-occlusive crises, the number of transfusions, low fetal hemoglobin, and hemolysis markers were the main determinants of these early markers of sickle cell nephropathy. These determinants could contribute to the early identification of high-risk patients and the improvement of screening strategies, enabling preventive and therapeutic interventions.

### Strengths of the study

This study assessed the relationship between early renal impairment and VOCs, hemolysis markers, and HbF in children. The use of the ACR is a methodological strength. Data from this study can inform health policies and strategies for preventing and managing sickle cell disease and its renal complications in pediatric populations in the DRC.

### Conclusion and outlook

This multicenter study highlights the persistence of a high prevalence of albuminuria and GHF in children with sickle cell disease in the DRC, suggesting a lack of significant progress in the prevention of sickle cell nephropathy over the past decade. These results underscore the need for early and systematic renal screening using simple and accessible tools such as the urinary ACR, particularly in resource-limited settings. GHF, which may represent the first detectable renal abnormality, and albuminuria should be considered complementary markers of early renal involvement. Since our data suggest that GHF precedes the onset of albuminuria, prospective interventional trials aimed at reducing hyperfiltration in early childhood are needed. Early identification of high-risk children could thus enable targeted management, including better access to hydroxyurea and enhanced renal monitoring, in order to delay progression to chronic kidney disease.

## Supplementary Information

Below is the link to the electronic supplementary material.

ESM 1 Graphical abstract (PPTX 503 KB)

## Data Availability

The data supporting the findings of this study are available from the corresponding author upon reasonable request**.**
